# Nutritional Profile and Carbohydrate Characterization of Spray-Dried Lentil, Pea and Chickpea Ingredients

**DOI:** 10.3390/foods2030338

**Published:** 2013-07-25

**Authors:** Susan M. Tosh, Edward R. Farnworth, Yolanda Brummer, Alison M. Duncan, Amanda J. Wright, Joyce I. Boye, Michèle Marcotte, Marzouk Benali

**Affiliations:** 1 Guelph Food Research Centre, Agriculture and Agri-Food Canada, Guelph, ON N1G 5C9, Canada; E-Mail: Yolanda.Brummer@agr.gc.ca; 2 Food Research and Development Centre, Agriculture and Agri-Food Canada, Saint-Hyacinthe, QC J2S 8E3, Canada; E-Mails: TedFarnworth@yahoo.com (E.R.F.); Joyce.Boye@agr.gc. (J.I.B.); Michele.Marcotte@agr.gc.ca (M.M.); 3 Department of Human Health and Nutritional Sciences, University of Guelph, Guelph, ON N1G 2W1, Canada; E-Mails: AMDuncan@uoguelph.ca (A.M.D.); AJWright@uoguelph.ca (A.J.W.); 4 Industrial Systems Optimization, Natural Resources Canada, Varennes, QC J3X 1S6, Canada; E-Mail: Marzouk.Benali@nrcan.gc.ca

**Keywords:** pulses, dietary fibre, peas, chickpeas, lentils

## Abstract

Although many consumers know that pulses are nutritious, long preparation times are frequently a barrier to consumption of lentils, dried peas and chickpeas. Therefore, a product has been developed which can be used as an ingredient in a wide variety of dishes without presoaking or precooking. Dried green peas, chickpeas or lentils were soaked, cooked, homogenized and spray-dried. Proximate analyses were conducted on the pulse powders and compared to an instant mashed potato product. Because the health benefits of pulses may be due in part to their carbohydrate content, a detailed carbohydrate analysis was carried out on the pulse powders. Pulse powders were higher in protein and total dietary fibre and lower in starch than potato flakes. After processing, the pulse powders maintained appreciable amounts of resistant starch (4.4%–5.2%). Total dietary fibre was higher in chickpeas and peas (26.2% and 27.1% respectively) than lentils (21.9%), whereas lentils had the highest protein content (22.7%). Pulse carbohydrates were rich in glucose, arabinose, galactose and uronic acids. Stachyose, a fermentable fibre, was the most abundant oligosaccharide, making up 1.5%–2.4% of the dried pulse powders. Spray-drying of cooked, homogenized pulses produces an easy to use ingredient with strong nutritional profile.

## 1. Introduction

Peas, chickpeas and lentils are recognized as nutritious sources of high quality plant-based protein [1,2]. Although much attention has been given to the amino acid content of pulses, they are also good sources of vitamins, minerals, phenolic compounds, dietary fibre and resistant and slowly digestible starch. 

Consumption of pulses on a regular basis has been associated with lower risks for the development of type 2 diabetes, coronary heart disease and some forms of cancer [3]. Those who consume pulses also tend to have lower rates of obesity and metabolic syndrome [4]. Therefore, a number of countries recommend that people consume pulses as part of a healthy diet [5,6].

However, long preparation times can be a barrier to pulse consumption. Chickpeas must be soaked before they are cooked and cooking times for chickpeas, peas and lentils can be in excess of an hour. Therefore, a powder made of cooked pulses which could be stored dry at room temperature and used directly in a wide variety of formulations has the potential to increase the market for pulses.

Processing of pulses changes their microstructure. Soaking and cooking gelatinize starch whereas shear disrupts the testa and potentially the cell walls exposing protein bodies and starch. This has the potential to change the digestibilities of the pulses and impact their functionalities. 

The purpose of this study was to examine the composition of the dietary fibre in innovative pea, chickpea, and lentil ingredients and compare them to a comparable product made from potatoes. Also, the microstructure of the pulse powders was compared to the original seeds.

## 2. Materials and Methods

### 2.1. Pulse Treatments

Pulses were obtained from Saskcan Pulse Trading Company, Rosetown, SK, Canada. Raw Kabuli chickpeas and green peas were soaked in water for 12 h at 4 °C. Large green Laird lentils were immersed in water and immediately processed. All wash and cooking water was retained and further processed. The chickpeas, lentils and green peas were then cooked in a 520-L steam cooking double-walled tank for 60, 30–45 and 90–105 min, respectively. They were mixed and homogenized (Urschel cutter and Microcut Stephan homogenizer) and then preheated in a vertical tubular heat exchanger (30–40 °C). The homogenized preheated material was then dried in a fast-spouted bed dryer with 4-mm spherical Teflon beads acting as inert intermediate drying medium to intensify the drying rate, thus reduce the dryer volume. The use of inert particles permits control of moisture content and size distribution of dried pulses. Inlet air temperature was varied from 180 to 195 °C. The solubility index and the final moisture content of dried pulses ranged from 20% to 27%, and from 2.3% to 6.5% (dry basis), respectively. The dried and powdered pulses were screened, sequentially, through 500 and 280 μm sieves, and then portioned into 100 g samples in individual semi-rigid high-density polyethylene opaque trays sealed under vacuum with a polyethylene film cover and stored at 4 °C. 

Commercial potato flakes (Dr. Oetker Classic Mashed Potatoes, Dr. Oetker Canada Ltd., Mississauga, ON, Canada) were similarly analyzed for comparison.

### 2.2. Proximate Analyses

Moisture content was determined by drying the samples at 80 °C in a vacuum oven until no further change in weight was observed. Protein content was determined by the Dumas combustion method (Leco FP-528, Mississauga, ON, Canada). The conversion factor used was *N* × 6.25 (AOAC method 997.02 [7]). Lipids were measured using a Soxhlet-type extraction in a Foss-Tecator extractor (Hillerød, Denmark) using hexane. Ash content was determined by charring the samples in an oven at 500 °C overnight. Starch analysis was done enzymatically. After hydrolysis with α-amylase and amyloglucosidase, glucose concentrations were measured spectrophotometrically with an automated glucose-oxidase assay (AOAC method 996.11 [7]). Dietary fibre was measured gravimetrically (AOAC method 991.43 [7]). This method does not measure oligosaccharides and under-estimates resistant starch, so these dietary fibre components were measured separately. 

### 2.3. Carbohydrate Analysis

The soluble sugar composition was studied using high-performance anion-exchange chromatography coupled with pulsed amperometric detection (HPAEC-PAD, Dionex, Sunnyvale, CA, USA) for sugar analysis [8]. Soluble sugars were extracted from chickpea, green pea and lentil treatment powders in 80% ethanol for 2 h. The extract volume was reduced in a rotary-evaporator and made to a known volume with water. This extract was analysed using HPAEC-PAD as outlined elsewhere [8] using glucose, sucrose, raffinose, stachyose and verbascose to prepare standard curves. Resistant starch was measured according to AOAC method 2002.02 [7] using a commercially available kit from Megazyme International (Bray, Ireland).

Sugar compositions of the soluble and insoluble fibre fractions were also analyzed. These fractions were extracted based on AOAC method 991.43 [7] for the measurement of total, soluble and insoluble dietary fibre in foods. Briefly, lentil, pea and chickpea powders were treated with thermostable α-amylase at 95–100 °C for 35 min in 2-(*N*-morpholino)ethanesulfonic acid/Tris buffer (0.5 M, pH 8.2). This was followed by protease (60 °C, 30 min) and amyloglucosidase (60 °C, 30 min, pH 4.1–4.8) digestions. Mixtures were boiled to deactivate enzymes and centrifuged to separate insoluble fibre. The soluble fibre was precipitated from the supernatant by the addition of 4 volumes of 95% ethanol and then separated and recovered by centrifugation. Both the insoluble and soluble fibre fractions were dried and ground before being reduced to their component sugars by acid hydrolysis. An accurately weighed portion (~20 mg) of fibre was treated with 72% H_2_SO_4_ for 1 h at room temperature (23 °C) followed by 3 h at 100 °C in 1 M H_2_SO_4_. Hydrolysates were diluted to 10 mL with water. They were further diluted until the concentration was within the standard curve and filtered prior to analysis. The ratio of sugars in the hydrolysates was analyzed by HPAEC-PAD as described for monosaccharide analysis in Brummer *et al.* [9]. Standard curves were prepared with rhamnose, arabinose, galactose, glucose, xylose, and mannose to determine concentrations.

Uronic acids are the negatively charged sugars which are found in pectins. The uronic acid content was determined by the colorimetric *m*-hydroxydiphenyl assay [8,10]. A standard curve was constructed with galacturonic acid monohydrate as the standard.

### 2.4. Microscopy

Microstructure of raw pulses and spray-dried pulse powders were studied using a Zeiss Axio imager A2 Microscope with a Zeiss Axiocam MRC5 camera (Zeiss, Oberkochen, Germany). Raw whole pulses were hydrated on wet filter paper in a covered petri dish overnight. They were sliced with a sharp blade and mounted on the slide in 50% glycerol and water. The spray-dried ingredient powders were mounted on a slide in 50% glycerol and water. General microstructure was studied using brightfield microscopy. Starch characteristics were determined using polarized light. To study the integrity of the cell walls, the structure was examined under fluorescent light (Zeiss filter set 38: excitation BP 470/40, beam splitter FT 495, and emission BP 525/50). Images were captured with Axiovision Software (Zeiss, Oberkochen, Germany).

### 2.5. Statistical Analysis

Statistical analyses were performed using Graph Prism v. 5.0 (GraphPad Software, La Jolla, CA, USA). Significant differences within data sets were determined using one-way or two-way analysis of variance (ANOVA) as required. Bonferroni [11] post-tests were conducted to compare means between treatments.

## 3. Results and Discussion

### 3.1. Proximate Analyses

A detailed chemical analysis was carried out on the spray dried powders. Emphasis was placed on carbohydrate composition because of the impact these constituents may have on the glycemic response and cholesterol lowering effects of pulses. The composition of the spray-dried pulses is shown in Table 1. Because the cook water was conserved and the soak water was added back before spray-drying, the differences between the composition of the raw and cooked products were small. The spray-dried powders contained approximately 1% more protein and 2% less starch than the corresponding raw pulses. The data in Table 1 reveal that the compositions of the chickpea, lentil and pea flours were similar to those reported previously by Dalghetty and Baik [12]. Within the pulse treatments, green pea had the highest proportions of resistant starch and oligosaccharides, while chickpea had the highest proportion of lipids and the lowest levels of oligosaccharides. Lentil had the highest proportions of protein, but the lowest proportions of sugars.

**Table 1:** Proximate and nutrient composition of dried treatment powders.

	Potato	Chickpea	Lentil	Green Pea	*n* ^ 1^
Energy (kcal/100 g) ^2^	299.8	282.7	263.7	245.2	
Moisture (%) ^3^	7.89 ± 0.10 ^a^	7.03 ± 0.17 ^b^	5.66 ± 0.05 ^d^	6.30 ± 0.08 ^c^	3
Protein (%)	5.4 ± 0.2 ^d^	18.3 ± 0.3 ^c^	22.7 ± 0.2 ^a^	21.3 ± 0.5 ^b^	4
Lipids (%)	0.24 ± 0.06 ^c^	4.97 ± 0.23 ^a^	0.70 ± 0.08 ^b^	0.60 ± 0.08 ^b^	3
Ash (%)	3.76 ± 0.15 ^a^	2.79 ± 0.07 ^b^	2.62 ± 0.06 ^b^	2.74 ± 0.23 ^b^	3
Digestible starch (%)	68.0 ±1.3 ^a^	40.2 ± 1.2 ^bc^	42.6 ± 1.0 ^b^	39.2 ± 1.3 ^c^	6
Sugars (%)	0.99 ± 0.00 ^c^	2.86 ± 0.56 ^a^	1.71 ± 0.04 ^b^	3.00 ± 0.36 ^a^	6
Total dietary fibre (%)	7.8 ± 0.6 ^c^	26.2 ± 2.7 ^ab^	21.9 ± 1.4 ^b^	27.1 ± 1.6 ^a^	4
Resistant starch (%)	0.78 ± 0.02 ^d^	4.44 ± 0.16 ^c^	4.75 ± 0.14 ^b^	5.17 ± 0.07 ^a^	9
Dietary fibre (%)	7.0 ± 0.6 ^c^	19.9 ± 2.5 ^a^	14.6 ± 1.1 ^b^	18.4 ± 1.0 ^ab^	3
Oligosaccharide (%)	ND ^4^	1.88 ± 0.09 ^c^	2.59 ± 0.07 ^b^	3.49 ± 0.59 ^a^	6

^1^ Number of samples contributing to mean; ^2^ Based on 100 g of dried powered material; ^3^ Data are expressed as means ± SD, means within a row followed by the same letter are not significantly different at *p* < 0.05; ^4^ ND, not detected.

The concentration of many important nutrients found in pulses can be greatly affected by soaking and cooking. Therefore, all soak water and cooking water was retained and included in the homogenized material. Taken together, the compositional analyses presented here show that, although pulses (dried peas, chickpeas and lentils) are often discussed as if they are homogeneous, there may be differences in such macronutrients as the carbohydrate fractions. The differences observed could lead to differential effects, particularly, in terms of influencing gut microbiota. For example, verbascose was not found in chickpeas, and since it is the largest molecular weight oligosaccharide detected, it may persist longer in the lower gut. Green peas had a higher ratio of insoluble to soluble fibre than chickpeas and lentils, which may influence the fermentation rate. 

**Figure 1:**
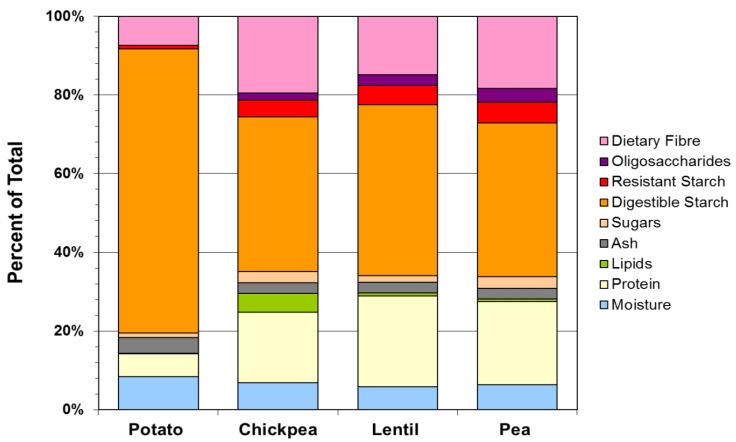
Representation of the composition of pulse ingredients and instant mashed potato product.

A general comparison of the pulse and potato ingredients is shown in Figure 1. Potato flakes were analyzed because they were used as a control in a human food-based trial conducted using these pulse powder ingredients herein described. Potato is commonly consumed, readily available in a dry powder commercial format, and has a carbohydrate composition that is different from the pulses under study. Compared to the potato powder, the pulse treatments were lower in digestible starch, and higher in protein, total lipids and dietary fibre. 

### 3.2. Carbohydrate Analysis

Table 2 shows the sugar composition of the carbohydrates in each dried treatment powder. Glucose, derived mainly from starch and cellulose (both composed entirely of glucose), makes up the largest part of the carbohydrate. The rhamnose, arabinose, galactose and uronic acids are derived mainly from the pectins. The sum of these four sugars was 10.8%, 9.3% and 9.5% for chickpeas, lentils, and peas, respectively. This suggests that chickpeas may be higher in pectin compared to the other pulses. The peas had the highest uronic acid content suggesting that the pectin in peas is more negatively charged than in other pulse treatments. The low levels of xylose (*i.e.*, 0.5% to 1.1%) suggest that the xyloglucans, which have a xylose backbone with glucose side chains, are relatively minor components of pulse carbohydrates. 

**Table 2:** Sugar composition of total carbohydrates expressed as a percentage of dried treatment powders.

	Potato	Chickpea	Lentil	Green Pea
Arabinose (%) ^1^	0.34 ± 0.02 ^c^	3.76 ± 0.19 ^a^	2.38 ± 0.16 ^b^	2.76 ± 0.13 ^b^
Galactose (%)	1.96 ± 0.17 ^b^	3.68 ± 0.20 ^a^	3.64 ± 0.22 ^a^	2.97 ± 0.31 ^ab^
Glucose (%)	72.9 ± 1.2 ^a^	46.9 ± 0.3 ^c^	50.5 ± 2.2 ^b^	50.2 ± 2.2 ^b^
Mannose (%)	ND^2^	ND	ND	ND
Rhamnose (%)	ND	ND	ND	0.29 ± 0.02
Uronic acids (%)	- ^3^	3.38 ± 0.37 ^a^	3.26 ± 0.24 ^a^	3.52 ± 0.16 ^a^
Xylose (%)	ND	0.50 ± 0.05 ^a^	0.83 ± 0.15 ^a^	1.10 ± 0.15 ^a^

^1^ Data are expressed as means ± SD (*n* = 6), means within a row followed by the same letter are not significantly different at *p* < 0.05; ^2^ ND, not detected; ^3^ Not analysed.

The sugar and oligosaccharide composition of the treatments are shown in Table 3. Glucose is the major sugar in potato, whereas pulses contain only small amounts of glucose, with sucrose being the major sugar present. The galacto-oligosaccharides are a family of short chain carbohydrates related to sucrose. The pea ingredient was found to contain the most total oligosaccharides (*i.e.*, 3.73% of total solids). Stachyose was the most abundant of the oligosaccharides, in agreement with previous research on cooked, soaked pulses [13]. Verbascose, the highest molecular weight galacto-oligosaccharide was not found in the chickpea powder. Similarly, others have reported the absence of verbascose in chickpeas, and higher levels of this compound in peas [13].

**Table 3:** Sugar and oligosaccharide composition of potato, chickpea, lentil and pea treatment powders, expressed as a percentage of the dried powders.

	Potato	Chickpea	Lentil	Green Pea
Sugars (%) ^1^	1.7	3.08	1.81	3.21
Sucrose (%)	ND ^2^	3.04 ± 0.57 ^a^	1.80 ± 0.04 ^b^	3.17 ± 0.37 ^a^
Glucose (%)	1.69 ± 0.01 ^a^	0.04 ± 0.03 ^b^	0.01 ± 0.01 ^c^	0.04 ± 0.01 ^b^
Oligosaccharides (%)	ND	2.02	2.75	3.73
Raffinose (%)	ND	0.53 ± 0.03 ^a^	0.32 ± 0.01 ^a^	0.48 ± 0.07 ^a^
Stachyose (%)	ND	1.49 ± 0.07 ^c^	1.79 ± 0.06 ^b^	2.36 ± 0.39 ^a^
Verbascose(%)	ND	ND	0.64 ± 0.01 ^b^	0.89 ± 1.17 ^a^
Total (%)	1.07	5.1	4.56	6.94

^1^ Data are expressed as means ± SD (*n* = 6), means within a row followed by the same letter are not significantly different at *p* < 0.05; ^2^ ND, not detected.

The distribution of neutral sugars found in the soluble and insoluble fractions of the dietary fibre are shown in Table 4. Once starch had been removed, the proportion of glucose was much smaller and the method was better able to detect mannose and rhamnose. For the neutral sugar fractions, peas had the highest ratio of insoluble to soluble fibre at 3.8:1, whereas the ratio in lentils was 2.7:1 and, in chickpeas, it was 2.2:1. The composition of the soluble fibre was similar for the different pulses. Green peas had lower levels of arabinose and glucose than chickpeas and lentils. Chickpeas had higher levels of galactose than lentil and green peas. The profile of the soluble fibres suggests that it is predominantly pectin. As expected, the glucose levels in the insoluble fibre were much higher due to the presence of cellulose. The lentil product had a higher proportion of xylose in the insoluble fibre fraction. The presence of rhamnose in the insoluble fraction suggests that not all of the pectin was solubilized.

### 3.3. Microscopy

**Figure 2:**
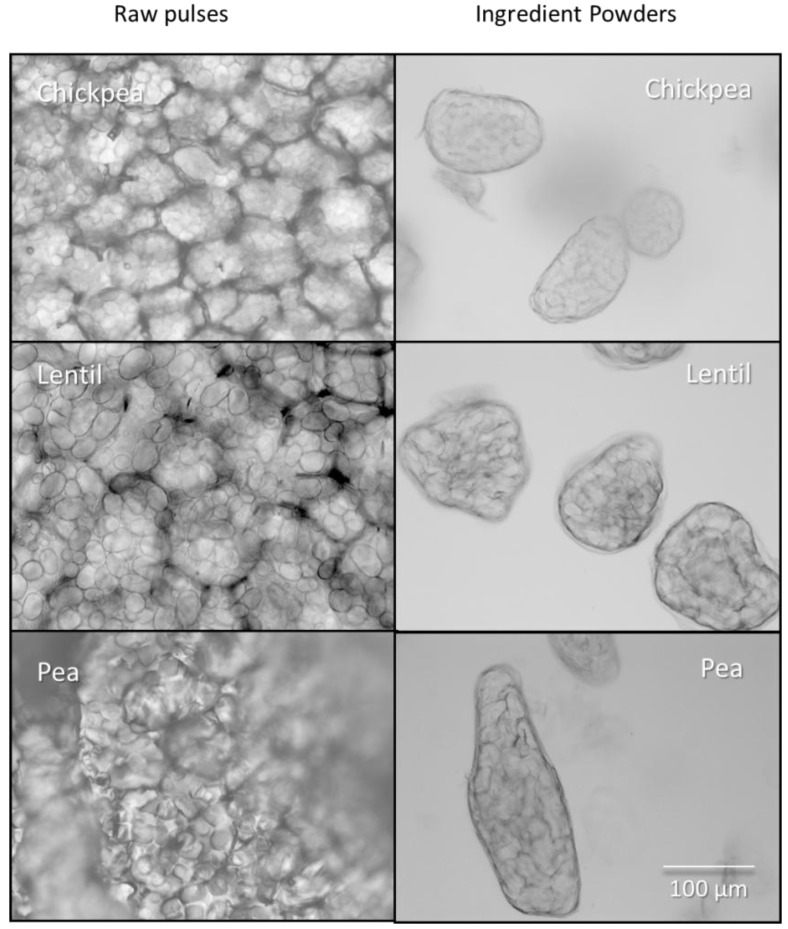
Bright field microscopy of raw pulses and spray-dried pulse ingredients.

**Figure 3:**
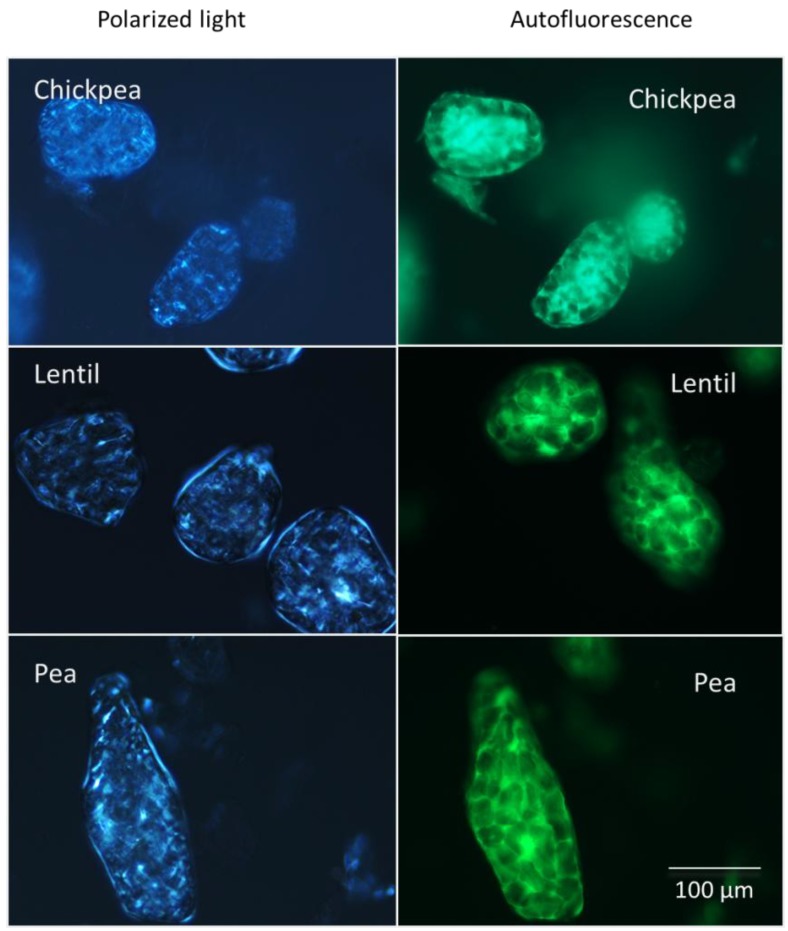
Polarized and autofluorescent images of spray-dried pulse powders. Under the polarized light ungelatinized starch is blue. Under light with a wave length of 470 nm cell walls fluoresce green.

Figure 2 shows the change in microstructure caused by processing. In the raw pulses, the starch granules are tightly packed into the cells and the cell walls are clearly visible. Individual cells appear to be arranged into groups of cells surrounded by a thicker layer of cell wall material. Under polarised light, the starch granules display Maltese crosses typical of ungelatinized starch (not shown). In the spray-dried pulse ingredients, the cell walls are intact and the starch granules are contained within the cells. Figure 3 shows the autofluorescence of the cell walls in the pulse ingredients. The starch granules are slightly swollen and they exhibit small shards of brightness rather than clear Maltese crosses under polarized light, showing that the starch has been partially gelatinized. It appears that the individual particles of the pulse powders are made up of the larger packets of cells distinguished in the intact pulses. 

**Table 4:** Neutral sugar distribution in the soluble and insoluble fibre fractions calculated as a percentage of total carbohydrates.

	Chickpea	Lentil	Green Pea
Soluble ^1^			
Arabinose (%) ^1^	6.74 ± 0.18 ^a^	6.31 ± 0.11 ^a^	5.58 ± 0.11 ^b^
Galactose (%)	4.32 ± 0.07 ^a^	2.81 ± 0.07 ^b^	2.55 ± 0.04 ^b^
Glucose (%)	5.58 ± 2.62 ^a^	6.32 ± 1.16 ^a^	2.81 ± 0.37 ^b^
Mannose (%)	2.18 ± 0.24 ^a^	2.32 ± 0.36 ^a^	2.12 ± 0.40 ^a^
Rhamnose (%)	1.23 ± 0.09 ^a^	0.56 ± 0.02 ^a^	0.85 ± 0.09 ^a^
Xylose (%)	0.52 ± 0.04 ^a^	1.17 ± 0.09 ^a^	1.03 ± 0.06 ^a^
Total (%)	20.6 ± 3.2	19.5 ± 1.8	14.9 ± 1.1
Insoluble ^1^			
Arabinose (%)	9.02 ± 1.9 ^a^	8.09 ± 0.23 ^a^	9.08 ± 1.07 ^a^
Galactose (%)	1.17 ± 0.15 ^a^	1.54 ± 0.05 ^a^	1.65 ± 0.08 ^a^
Glucose (%)	33.5 ± 8.6 ^c^	36.4 ± 0.6 ^b^	40.7 ± 6.13 ^a^
Mannose (%)	0.69 ± 0.06 ^a^	0.57 ± 0.19 ^a^	0.76 ± 0.06 ^a^
Rhamnose (%)	0.45 ± 0.07 ^a^	0.48 ± 0.06 ^a^	0.57 ± 0.10 ^a.^
Xylose (%)	1.10 ± 0.27 ^b^	4.56 ± 0.37 ^a^	1.10 ± 0.15 ^b^
Total (%)	46.0 ± 11.1	51.6 ± 1.5	57.2 ± 8.7

^1^ Data are expressed as means ± SD (*n* = 6 for soluble fibre, *n* = 18 for insoluble fibre), means within a row followed by the same letter are not significantly different at *p* < 0.05.

### 3.4. Relation to *in Vivo* Data

The resistant starch fraction of pulses is important because is not hydrolysed in the small intestine, and is therefore available to be fermented by the colonic microflora. Microscopical examination of the pulse ingredients showed that the cell walls remained intact and starch enclosed within the cells was only partially gelatinized. This structure is very different from flours, where the cell walls are shattered, exposing the starch granules and protein bodies [14]. Raffinose, stachyose and verbascose [15], are soluble carbohydrates which are not affected by digestive enzymes secreted in the upper digestive tract. They pass to the colon intact where they are available for fermentation [16]. Some metabolites of the bacteria of the gastrointestinal tract, such as the short chain fatty acids acetate, propionate, and butyrate [3,16], are beneficial. Other by-products such as gases lead to flatulence sometimes associated with pulse consumption [16]. The high proportions of dietary fibre, resistant starches, and oligosaccharides found in the three pulses in this study would be expected to favour the production of short chain fatty acids and gases in the colon. However, as previously reported, the incidence and severity of flatulence and intestinal discomfort following daily consumption of 100 g of each pulse powder were minor and short in duration [17]. Similarly, according to analysis of participant fecal samples, there were no significant changes in fecal short chain fatty acid content or in the colonic microbial population as a result of consuming the pulse ingredients [18].

A recent study [19], investigated the effect of a pulse-rich diet on the risk factors for cardiovascular disease in older adults. Individuals with mild hypercholesterolemia consumed pulse-based foods twice a day and after two months their serum LDL-cholesterol levels were reduced by 7.9%. However, when a group of healthy participants consumed the pulse ingredients described here for 28 days, there were no significant reductions in serum cholesterol levels [20]. However, the participants easily incorporate the reconstituted pulse powder into their daily diets without any problems. There also were no dropouts due to diet fatigue, dislike or gastrointestinal effects [17]. The participants were young (19–40 years of age), healthy and did not have elevated baseline cholesterol or glucose levels which may have impacted the ability to detect any reductions in cholesterol or fasting glucose levels [20]. 

Other researchers have found variable results for processed pulse products in terms of metabolic response. In another study, pinto bean, black-eyed pea and navy bean pastes did not cause significant reductions in post-prandial glucose response [21]. On the other hand, there is consistent evidence that consumption of whole pulses lowers postprandial blood glucose [22,23,24]. The apparent discrepancies may be a result of changes in microstructure of the pulses during processing, such as cell wall disruption and reductions in resistant starch, which could increase the rate of starch digestion.

## 4. Conclusions

A variety of pulses were processed into easily rehydratable powders using spray-drying technology. A thorough carbohydrate composition analysis revealed differences in their nutritional profiles. The human study showed there was no effect on cholesterol, fasting glucose or fecal endpoints, highlighting the need to consider more microstructural matrix effects. Taken all together, it would appear that the production of spray-dried, cooked pulse ingredients is convenient and nutritious. More research is needed to ascertain the effects of food processing techniques on the health benefits provided by pulse food ingredients.

Although the composition of the macronutrients in the pulse ingredients tested was similar, some significant differences were observed. Of note were the higher levels of lipid in chickpeas and protein in lentils. There were also differences in the oligosaccharide profiles and the ratios of sugars which make up the soluble and insoluble fibre fractions. This study contributes to the available information concerning the composition of pulse fibres. Further research into nutritionally-relevant pulse varieties may be useful in understanding the way that pulses deliver health benefits.
